# Crack-cocaine use practices, harms and respiratory problems: an analysis of gender differences using data from a cross-sectional survey in England

**DOI:** 10.1186/s12889-026-27073-1

**Published:** 2026-04-02

**Authors:** Vivian D. Hope, Caitlynne McGaff, Casey Sharpe, Sujit D. Rathod, Lucy Platt, Jenny Scott, SIPP Project Team, Joanna Busza, Sedona Sweeney, Lorna Guinness, Cedomir Vuckovic, Ian Yoon, Alexandre Piot, Andrew Preston, Niamh Eastwood, Louise Wilkins, Shoba Ram, Catherine Lord, Philippe Bonnet, Peter Furlong, Magdalena Harris

**Affiliations:** 1 Public Health Institute and School of Public and Allied Health, Liverpool John Moores University, 3rd Floor Tithebarn Building, 79 Tithebarn Street, Liverpool, L2 2ER UK; 2 Department for Public Health, Environments and Society, London School of Hygiene & Tropical Medicine, 15-17 Tavistock Place, London, WC1H 9SH UK; 3 Centre for Academic Primary Care, Population Health Sciences, Bristol Medical School, University of Bristol, Canynge Hall, 39 Whatley Road, Bristol, BS8 2PS UK

**Keywords:** Crack-cocaine, Smoking, Respiratory problems, Gender

## Abstract

**Background:**

The use of stimulants, such as crack-cocaine, is a global public health concern. Crack-cocaine use is increasing in the UK, but available data is focused on those who inject or also use opioids. To address this gap, characteristics of people using crack-cocaine in England, including respiratory problems among those who smoke, and variation in these by gender are described.

**Methods:**

Adults self-reporting crack-cocaine use in the past 28 days, recruited by specialist services and peer networks in six sites during 2023, completed a self-report survey about demographic characteristics, drug use, crack use practices, health problems and service use. Bivariable analyses and logistic regression were used to explore gender-related differences in crack-cocaine use and crack-related respiratory problems.

**Results:**

The participants’ (*n* = 731) median age was 42 years and 71% were men. Overall, 54% were stably housed, 71% had ever been imprisoned and 28% reported emergency department attendance in past 6 months. In the past 28 days, 99% had smoked crack-cocaine (44% shared pipes), with 30% injecting crack. Poly-sedative use was common including heroin (78%), pregabalin/gabapentin (41%), and benzodiazepines (28%), with 62% receiving opioid substitution therapy. Use of drugs normally smoked was common (90% tobacco, 62% cannabis and 25% spice). Women reported less polydrug use but more often vaped nicotine. Crack-related respiratory symptoms among those smoking crack were reported by 67% of women and 58% of men. In both men and women these symptoms were associated with increasing time since first crack-cocaine use and pregabalin/gabapentin use. In men they were also associated with food insecurity; smoking tobacco; temporary employment; and use in abandoned buildings or at friend’s place; reduced odds were associated with current heroin use and using with a close friend. Among women, having a respiratory symptom was also associated with sharing pipes.

**Conclusions:**

Respiratory health problems are common among those smoking crack-cocaine, particularly among women. In combination with high poly-sedative use, this poses a mortality risk from respiratory depression. UK service provision is focused on prevention of opiate and injection-related risks. Services for people who use crack-cocaine and low-threshold respiratory care pathways require prioritisation to reduce avoidable morbidity and mortality.

## Background

The health issues associated with the smoking or injection of stimulants – such as blood borne viral transmission, sexually transmitted infections, respiratory conditions, and mental health problems – are a global public health concern [1–4]. Cocaine is a stimulant drug that predominantly comes in two forms: as a powder or as a crystal-like solid (or ‘rock’) known as crack. Both forms can be injected, but only crack-cocaine can be smoked, with powder cocaine generally administered through intranasal insufflation (snorting) [5]. In the United Kingdom cocaine is the most common stimulant used on the unregulated market, with crack-cocaine predominantly used by more marginalised populations than powder cocaine [6]. England has the highest prevalence of crack-cocaine use in Europe [7], with the reported number of people using crack-cocaine increasing from 144,558 in 2016–17 to 176,752 in 2019–20 [8]. Opioid use (mostly heroin) and crack-cocaine use together is common in England, either through injection in a combination solution, injection of heroin and inhalation of crack-cocaine, or inhalation of both substances separately [9]. Despite the increase in the number of people who use crack-cocaine, there is very little known about people who primarily or only smoke crack-cocaine, as drug use monitoring systems tend to focus on opioids or injection drug use [10–12]. However, modelling data suggest that around one-third of people who use crack-cocaine do not use heroin or are not on opioid substitution therapy (OST) [13] and treatment entry numbers for people who use crack-cocaine but not opioids have increased from 4,509 in 2012–13 [14] to 12,283 in 2024–25 [6].

In England established harm reduction interventions for those who inject drugs or who use opioids are widely available. Needle and syringe programs (NSPs) and OST provision are evidenced to reduce blood borne virus (BBV) transmission [15, 16]. OST reduces illicit opioid use, injection frequency, and all-cause mortality [17–19]. NSPs provide harm reduction advice, which reduces fatal overdose risk, and distribute sterile injecting equipment which reduces the risk of BBV acquisition [15, 20], skin and soft tissue infections and associated sequelae (systemic infections such as septicaemia and endocarditis) [21].

In England specialist services for people who use drugs typically provide OST and/or NSP in line with relevant guidance [22, 23]. NSP is also delivered in some pharmacies. NSP provision is facilitated through listed exemptions for injecting equipment (e.g., needles, syringes, filters) under Sect. 9 A of the Misuse of Drugs Act (MDA), 1971 [24], with the addition of foil to facilitate heroin smoking in 2014. The provision of safer inhalation equipment for stimulant use is however, prohibited as it is not currently included as an exemption under the MDA. A consequence of this prohibition of pipe provision is the use of less safe homemade or repurposed implements to smoke crack-cocaine [9]. Unlike OST provision for opioid dependency, there are no stimulant agonist therapies currently licensed for provision in the UK. This leaves specialist drug services with little to offer to people who smoke crack-cocaine apart from psychosocial interventions which are not always desired, available, or appropriate [9]. Services in England are, therefore, more orientated to meet the needs of people who inject drugs and/or use opioids and engage them with ancillary interventions and support such as housing and other social service referrals, sexual health and BBV screening [17, 20]. People who smoke crack-cocaine may have little reason to disclose their use or present to services. This limits the supports available to them and their visibility as a group in need. Given UK research and public health monitoring tends to focus on injecting practice and recruits through specialist drug services, little is also known about the characteristics of people who use crack-cocaine, particularly those that smoke, their drug use practices, or health risks and needs.

Global evidence indicates that women may experience greater harm than men from the use of illicit drugs, particularly among those who inject or use heroin [25–29]. Women can, for example, experience higher risk of infections (e.g., HIV and hepatitis C) [25, 26] and injuries [28], and there are gender differences in mortality [27, 29] as men consistently have higher crude rates of mortality whereas women have higher standardised rate [29]. These differences are likely to be underpinned by a range of factors: women using drugs may experience greater marginalisation and stigma, gender-based violence, use of drugs procured by others, and engage in sex work more than men [30–33]. However, there is only limited evidence of gender-related differences in harm among people who use crack-cocaine [34–36].

Initial evidence, mostly from Canada, indicates that provision of safer crack-cocaine smoking equipment can help reduce crack-related health harms, injecting and increase engagement with social and healthcare services [37–42]. International evidence may not be generalisable to the UK, given variation in local cultures of drug use and administration practices. To address gaps in UK knowledge around crack-cocaine use and crack-cocaine inhalation-related harms we describe the socio-demographic characteristics, crack-cocaine use practices, and self-reported respiratory harms among this population, using gender as analytic frame. Thus, this paper describes the characteristics, drug use practices, respiratory symptoms and health outcomes of adults who use crack in England and how these vary by gender.

## Methods

### Safe Inhalation Pipe Provision (SIPP)

The Safe Inhalation Pipe Provision (SIPP) project is a mixed-methods evaluation of a crack inhalation equipment and workforce training intervention at six sites in England, to generate evidence of intervention feasibility, acceptability, outcomes and impact. The quasi-experimental impact evaluation included pre- and post-intervention surveys at intervention and control sites. Full methodological detail is provided in the published protocol [43]. Data used in this analysis are from the SIPP project pre-intervention baseline survey.

### Data collection

Baseline survey participants were recruited in three intervention and three control sites through specialist drug services, one sex worker support service (in one intervention site) and three peer networks. Peer networks operated at one control and two intervention sites, with a focus on recruiting and conducting surveys with people who used crack-cocaine and did not engage with drug services. Baseline survey data were collected between February to July 2023. The services and peer networks were allocated recruitment targets in approximate proportion with the study site population and size of the service client base.

Participants were eligible if they were aged 18 years or older, spoke English, self-identified crack-cocaine use (injecting or smoking) in the past 28 days and had capacity to provide informed consent. Service recruitment initially focused on a list of clients randomly generated from a list of all crack-using clients at each service. At week eight, recruitment was expanded to include key worker referral, client ‘walk in’s’, outreach service provision, and BBV screening sessions. This expansion was to overcome the challenges of reaching clients on the random list within a short time-frame, particularly by phone (given frequency of phone number changes, reluctance to answer calls from services, etc.). Providers screened potential participants through client records, to ascertain crack-cocaine use was listed, and verbally, to confirm recent use. Surveys were conducted on Android tablets using Open Data Kit (V2024.1) and were administered by a drug treatment service staff member, a peer volunteer, or self-administered by the participant if preferred.

Peer recruitment was facilated by people with current or former experience of crack-cocaine use, utilising three overlapping approaches: 1) peer-initiated recruitment through personal networks of people who use crack cocaine, 2) via street outreach, and 3) through outreach by peers working with a hepatitis C partner organisation. The inclusion of peer recruitment was to access populations not, or not regularly, engaged with specialist drug services and all peers were supported by the research team including through the provision of training and supervision.

Baseline survey questions were close-ended and items were developed from measures used in comparable questionnaires of people who use crack-cocaine in Canada [4, 37, 38, 40, 44, 45] and a brief exploratory questionnaire, developed by the senior author and conducted by peers in 2020. Detail about the questionnaire development is included in the protocol [43]. Interviewers first screened for eligibility, took informed consent, and started the questionnaire. The questionnaire included the following sections: socio-demographic characteristics (including one question on gender and one on trans identification), drug use, routes of administration, crack-cocaine use practices (routes of crack-cocaine administration, sharing pipes, use of homemade pipes, crack-cocaine use locations and companions), contact with the criminal justice system, respiratory-related diagnoses (i.e., ever been diagnosed with asthma, tuberculosis, emphysema or chronic obstructive pulmonary disease (COPD)) and hospitalisations (i.e., ever been in hospital for COPD, lung cancer, pneumonia, pneumothorax, asthma, bronchitis, or crack lung), and engagement with social and healthcare services, including drug specialist services. Participants who had smoked crack in the past 28 days were asked: “*In the past 28 days, has smoking crack caused you any of the following problems?*” with the answer options of “*Cut/burned mouth*”, “*Cut/burned fingers*”, “*Breathing problems*”, “*Chest pain*”, “*Coughing up blood/phlegm*”, “*Paranoia/hallucinations*”, and “*Anxiety/Depression*”. Those reporting a breathing problems, chest pain, and/or coughing up blood/phlegm were coded as having ‘symptoms of a crack-related respiratory problem’.

### Analysis

First descriptive statistics are used to describe participants socio-demographic characteristics; drugs use patterns and practices; utilisation of health, support and welfare services; contact with criminal justice; and health outcomes. For respiratory health related outcomes, analysis was restricted to those who reported smoking crack-cocaine, either as the only route of administration or in addition to injecting. Variation in these by gender identification (i.e., men include cis and trans men; women are cis or trans women) are explored using Chi-Square test for categorical variables (Pearson, with Fisher's Exact Test, used for two-by-two tables where there were cells with expected counts < 5) and for continuous variables normality was assessed using the Kolmogorov–Smirnov test with the T-test used for those normally distributed and Mann–Whitney U for those not.

Finally, bivariable analyses (Chi-Square, T-test or Mann–Whitney U) were used to explore associations between covariates and reporting symptoms of a crack-related respiratory problem among those who reported smoking crack-cocaine for each gender. The focus of this analysis was to identify factors that those working with people who use drugs could use to inform the targeting of interventions. Thus, the variables used in these analyses were social demographic characteristics and drug use related practices and behaviours. Those variables associated with having symptoms of a crack-related respiratory problem (*p* < 0.1) in the bivariable analyses were then entered into a logistic regression model for each gender (using the forward stepwise procedure with inclusion access using the likelihood ratio test, *p* > 0.05). Analyses were undertaken in SPSS version 29.

## Results

In total 733 participants completed the baseline survey. Two were excluded from this analysis as one declined to identify their gender and other identified as non-binary, giving a total sample of 731.

### Socio-demographics

Participant characteristics are given in Table 1 (*N* = 731 unless otherwise stated, values less than this are due to participants not responding to specific questions): their median age was 42 (IQR 37–47) years, 71% (*n* = 520) of participants were men, 13% (96/728) were mixed ethnicity or an ethnic minority, and 10% (74/719) identified as gay, lesbian or bisexual. Most participants were cis-gendered, with 1.1% (8/729) trans-gender.

**Table 1:** Socio-demographic characteristics by gender

		**Gender**				*All*		*p*
		**Men**		**Women**				
*Total*		*520*	*71%*	*211*	*29%*	*731*	*100%*	
*Social demographics*								
Age, years	Mean	42.4		41.2		42.1		
	Median	42		41		42		0.024^‖^
	Range (Q25, Q75)	53 (37, 48)		42 (36, 46)		53 (37, 47)		
Ethnicity~	Minority	68	13%	28	13%	96	13%	0.941
	White	450	87%	182	87%	632	87%	
	Total	518		210		728		
Sexual orientation*	Heterosexual	493	96%	152	74%	645	90%	<0.001
	Gay, lesbian, bisexual or in another way	21	4%	53	26%	74	10%	
	Total	514		205		719		
Housing status	Homeless/rough sleeper	101	19%	30	14%	131	18%	0.197
	Unstable ^†^	131	25%	52	25%	183	25%	
	Stable ^‡^	271	52%	125	59%	396	54%	
	Other/refuse	17	3%	4	2%	21	3%	
How did you get by in the past 6 months?	Held a regular job (full or part-time)	31	6%	10	5%	41	6%	0.515
	Benefits	464	89%	192	91%	656	90%	0.476
	Temporary work	58	11%	11	5%	69	9%	0.013
	Borrowed/got money from family or friends	242	47%	102	48%	344	47%	0.658
	Sold sex ^§^	6	1%	64	30%	70	10%	<0.001
	Sold drugs	76	15%	37	18%	113	15%	0.322
	Theft/burglary	107	21%	47	22%	154	21%	0.610
	Begged	172	33%	60	28%	232	32%	0.222
	Other(s)/None of the above	22	4%	6	3%	28	4%	0.376
Can you always access food when you need it?		377	73%	146	70%	523	72%	0.459
	Total	517		208		725		
*Contact with* *criminal justice system*								
Have you ever been in prison or a young offenders' institution?		402	78%	115	55%	517	71%	<0.001
	Total	517		208		725		
In the last six months, did the police stop and search you?		212	41%	67	32%	279	38%	0.032
	Total	518		207		725		
In the last six months, did the police take away your smoking kit or any other drug equipment?		107	21%	23	11%	130	18%	0.003
	Total	518		206		724		

A total is given for an item when this differs from 731 due to people declining to answer, or when the data relates to a subgroup

~ Ethnic minority refers to all ethnic groups excluding White only ethnicities. These include Asian/British Asian, Black/Black British, Arah, Hispanic/Latino, and ‘other’ ethnicities

† Unstable includes hostel, squat, prison, rehab, staying friends & family ('sofa surfing').

‡ Stable includes having own place, and being in a house share

§ Sold sex, any sexual services i.e. oral, vaginal, anal, etc

‖ Mann-Whitney U test

* Gay, lesbian, or bisexual includes 4 reporting other sexual orientations; 12 missing (don't know=6, declined to answer=6).

Reports of unstable housing were common, with 18% of participants currently homeless (e.g., sleeping on the street) and 25% in unstable accommodation (e.g., living a hostel, ‘sofa surfing’, squat, etc.). When asked about sources of income in the past 6 months, 101 (14%) reported having a job (full or part-time job or temporary work), with 90% receiving benefits (universal credit). Overall, 257 (35%) reported a criminalised or illicit form of income generation, including theft/burglary (21%), selling drugs (15%), and/or sex work (10%). Over a quarter (28%, 202/725) could not always access food when they need it.

Contact with the criminal justice system was common, most of the participants reported ever being imprisoned (71%, 517/725). In the last six months, 38% had been stopped and searched by the police (279/725), and 18% had had their smoking kit or other drug equipment confiscated by police (130/724).

### Crack-cocaine use

The median age of first use of crack-cocaine was 22 years (IQR 17–30) and the median time since first use of crack-cocaine was 21 years (IQR 12–27; Table 2). Almost all (99%, 725/731) smoked crack-cocaine in past 28 days, and 30% (220/731) had injected it (6 participants reported using crack-cocaine only by injection), with daily crack-cocaine use reported by 56%. Those injecting crack-cocaine in past 28 days reported doing this on a mean of 16 days, with 94% injecting crack-cocaine with heroin (207/220). The use of homemade pipes for crack-cocaine smoking was reported by 69% (500/725), and the sharing of pipes was reported by 44% (320/725).

**Table 2:** Drug use by gender

		**Gender**				*All*		*p*
		**Men**		**Women**				
*Total*		*520*	*71%*	*211*	*29%*	*731*	*100%*	
*Crack use*								
How old were you when you first started using crack? (years)	Mean	21.8		23.9		23.9		
	Median	20		22		22		0.003^‖^
	Range (Q25, Q75)	50 (16, 26)		45 (17, 30)		45 (17, 30)		
Time since first crack use, in years	Mean	20.6		17.6		19.7		
	Median	22		19		21		<0.001^‖^
	Range (Q25, Q75)	51 (13, 28)		42 (9, 25)		51 (12, 27)		
	Total	519		207		726		
Smoked or injected crack daily, past 28 days		280	54%	126	60%	406	56%	0.148
Frequency of crack use past 28 days, in days	Mean	21.0		22.3		21.4		
	Median	28		28		28		0.100^‖^
	Range (Q25, Q75)	27 (14, 28)		27 (20, 28)		27 (15, 28)		
Smoked crack, past 28 days		515	99%	210	100%	725	99%	0.679^¶^
Injected crack, past 28 days		164	32%	56	27%	220	30%	0.182
Among those injecting past 28 days, days injecting crack	Mean	16.6		14.7		16.1		
	Median	19.5		13		16		0.307^‖^
	Range (Q25, Q75)	27 (5, 28)		27 (4, 28)		27 (4, 28)		
Injected crack with heroin in past 28 days (among all participants)		155	30%	52	25%	207	28%	0.160
Used homemade pipe, among those smoking past 28 days.		363	70%	137	65%	500	69%	0.166
	Total	515		210		725		
Shared pipes, among those smoking past 28 days.		228	44%	92	44%	320	44%	0.909
	Total	515		210		725		
Places where crack was used past 28 days (among those smoking past 28 days)	At home	372	72%	161	77%	533	74%	0.220
	At work	11	2%	6	3%	17	2%	0.591^¶^
	At a friend's place	177	34%	100	48%	277	38%	0.001
	Outdoors	266	52%	110	52%	376	52%	0.858
	In a semi-public space (e.g. stairwell)	156	30%	68	32%	224	31%	0.581
	Abandoned building	121	23%	43	20%	164	23%	0.378
	Somewhere else	45	9%	26	12%	71	10%	0.134
	Can't remember/don't know where	6	1%	5	2%	11	2%	0.312^¶^
	Total	515		210		725		
Smoked crack with (among those smoking past 28 days):	Partner	87	17%	84	40%	171	24%	0.000
	Other family member	39	8%	18	9%	57	8%	0.650
	Close friend	192	37%	69	33%	261	36%	0.260
	Housemates/co-residents	63	12%	30	14%	93	13%	0.453
	Dealer/runner	23	4%	20	10%	43	6%	0.009
	Casual friend/acquaintance	224	43%	86	41%	310	43%	0.530
	Client (e.g., paying sex partner)	3	1%	12	6%	15	2%	<0.001^¶^
	Sex worker	14	3%	8	4%	22	3%	0.437
	Someone else	20	4%	12	6%	32	4%	0.276
	Can't remember/don't know	5	1%	2	1%	7	1%	1.000^¶^
	Total	515		210		725		
On typical day, how much did you spend on crack, in pounds (£s)?	Mean	102.5		99.9		101.7		0.899
	Median	40		40		40		0.457^‖^
	Range (Q25, Q75)	2,998 (20, 100)		2,000 (20, 80)		3,000 (20, 89)		
	Total	470		191		661		
*Other* *drugs/substances*								
Substances used past 28 days	Heroin	403	78%	165	78%	568	78%	0.837
	Cannabis/weed	341	66%	112	53%	453	62%	0.002
	Pregabalin/gabapentin	205	39%	98	46%	303	41%	0.081
	Benzodiazepines	147	28%	56	27%	203	28%	0.636
	Cocaine	152	29%	44	21%	196	27%	0.021
	Spice/synthetic cannabinoids	156	30%	30	14%	186	25%	0.000
	Amphetamines (speed)	56	11%	10	5%	66	9%	0.010
	Ketamine	41	8%	13	6%	54	7%	0.420
	Mephedrone/M-cat	37	7%	16	8%	53	7%	0.825
	Tramadol	35	7%	15	7%	50	7%	0.854
	Crystal meth	18	3%	2	1%	20	3%	0.059
	GHB/GBL/G	11	2%	4	2%	15	2%	1.000^¶^
	Alcohol	302	58%	120	57%	422	58%	0.765
	Tobacco	470	90%	189	90%	659	90%	0.739
	Vapes/E-cigs	199	38%	99	47%	298	41%	0.031
Injected drug past 28 days		175	34%	65	31%	240	33%	0.457

A total is given for an item when this differs from 731 due to people declining to answer, or when the data relates to a subgroup

‖ Mann-Whitney U test

¶ Chi-squared using Fisher's Exact Test.

The most commonly reported place to smoke crack-cocaine during the past 28 days was ‘at home’ (73%), and half (51%) reported smoking crack-cocaine outdoors (Table 2). Smoking crack-cocaine with casual friend/acquaintances was reported by 42%, with close friends by 36% and with a partner by 23%. The median estimated expenditure on crack-cocaine on a typical day was £40 (IQR £20–89, *n* = 661).

### Use of other substances

The use of other drugs in past 28 days was common with 78% reporting use of heroin, and 62% cannabis (Table 2). The use of pregabalin and/or gabapentin (41%), benzodiazepines (28%), and cocaine (27%) was also common. One-third (32%, 236/731) reported using four or more other illicit drugs, in addition to crack-cocaine, in past 28 days, with only 26 (3.5%) reporting no illicit drug use other than crack-cocaine. Women reported using fewer of the other drugs asked about than men (women median 2, IQR1-4; men median 3 IQR2-4, *p* = 0.01). Overall, 42% (309/731) reporting using heroin and pregabalin/gabapentin or benzodiazepines, with 17% (127) reporting using heroin, pregabalin/gabapentin and benzodiazepines.

Injection of non-prescription drugs in the past 28 days was reported by 33% (*n* = 240). The drug most commonly injected was heroin: 94% (222/237) of those injecting had injected heroin in past 28 days. Overall, 47% (346/731) reported that they had used heroin in the past 28 days but did not report injecting, suggesting they likely smoked heroin. The use of alcohol (58%) and vaping (41%) was common and the use of drugs which are normally smoked was extensive (90% tobacco, 62% cannabis, and 25% spice/synthetic cannabis; Table 2).

### Health issues

Participants reported a range of health issues that they related to their crack-cocaine use (Table 3). Among people who smoked crack-cocaine, 83% (*n* = 600/721) reported a symptom of a respiratory problem, physical harm or mental health issue in the past 28 days that they related to their crack use. Over half reported ‘anxiety or depression’ (57%, 412/721), with 44% (317/721) reporting breathing problems, and 41% (293/721) cut or burned fingers (Table 3). Overall, 60% (435/721) reported one or more of three respiratory symptoms that they were asked about in relation to their crack-cocaine use (i.e., problem with breathing, chest pain, coughing blood or phlegm).

**Table 3:** Health issues and service use by gender

		**Men**		**Women**		**All**		*p*
		520	71%	211	29%	731	100%	
*In the past 28 days, has smoking crack caused you any of the following problems? (among those smoking crack in past 28 days):*	Breathing problems	206	40%	111	54%	317	44%	0.001
	Chest pain	162	32%	66	32%	228	32%	0.924
	Coughing blood or phlegm	176	34%	76	37%	252	35%	0.529
	*Any of the above crack related respiratory problems*	*297*	*58%*	*138*	*67%*	*435*	*60%*	*0.027*
	Cut or burned mouth	131	25%	59	29%	190	26%	0.406
	Cut or burned fingers	204	40%	89	43%	293	41%	0.413
	Paranoia or hallucinations	186	36%	75	36%	261	36%	0.991
	Anxiety or depression	290	56%	122	59%	412	57%	0.537
	*None of the above*	*90*	*18%*	*31*	*15%*	*121*	*17%*	*0.410*
	*Total*	*514*		*207*		*721*		
*Ever been in hospital for:*	Chronic obstructive pulmonary disease (COPD)	28	5%	17	8%	45	6%	0.170
	Lung cancer	3	1%	2	1%	5	1%	0.629^¶^
	Pneumonia	74	14%	51	25%	125	17%	0.001
	Pneumothorax	3	1%	2	1%	5	1%	0.629^¶^
	Asthma	56	11%	22	11%	78	11%	0.901
	Bronchitis	26	5%	14	7%	40	6%	0.374
	Crack lung	20	4%	14	7%	34	5%	0.103
	*Any of the above*	*144*	*28%*	*78*	*38%*	*222*	*31%*	0.012
	COVID-19	25	5%	10	5%	35	5%	0.975
	Influenza	14	3%	4	2%	18	2%	0.532
	Other	37	7%	9	4%	46	6%	0.153
	*None of the above*	315	61%	111	53%	426	59%	0.050
	*Total*	*514*		*208*		*722*		
*Ever diagnosed with:*	Asthma	145	28%	77	36%	222	30%	0.022
	Blood Poisoning (septicaemia, endocarditis)	68	13%	30	14%	98	13%	0.682
	Emphysema or COPD	52	10%	32	15%	84	11%	0.047
	Hepatitis C	172	33%	54	26%	226	31%	0.047
	HIV	9	2%	2	1%	11	2%	0.738^¶^
	Tuberculosis (TB)	10	2%	5	2%	15	2%	0.774^¶^
	None of the above	229	44%	87	41%	316	43%	0.488
*Services used past 6 months:*	Seen a General Practitioner	321	62%	138	65%	459	63%	0.352
	Outreach nurse	149	29%	65	31%	214	29%	0.562
	Dentist	65	13%	30	14%	95	13%	0.531
	Been to Accident & Emergency	135	26%	68	32%	203	28%	0.087
	Used an ambulance	82	16%	52	25%	134	18%	0.005
	Been admitted to hospital	93	18%	61	29%	154	21%	0.001
	Drug Treatment Service including street outreach	367	71%	141	67%	508	69%	0.318
	Needle & Syringe Programme (NSP)	178	34%	61	29%	239	33%	0.165
	Day centre/food bank/street soup kitchen	272	52%	115	55%	387	53%	0.590
	Support service for sex workers	5	1%	47	22%	52	7%	0.000
	Job Centre	180	35%	68	32%	248	34%	0.537
	None of the above	27	5%	4	2%	31	4%	0.045
Are you taking prescribed maintenance drug treatment, i.e., opioid substitution therapy?		334	64%	116	55%	450	62%	0.020
	*Total*	*518*		*210*		*728*		

A total is given for an item when this differs from 731 due to people declining to answer, or the data relates to a subgroup

¶ Chi-squared using Fisher's Exact Test

Two-fifths (41%, 295/722) reported ever being hospitalised for a respiratory-related health problem, with pneumonia being the most common reason for this (17%), followed by asthma (11%, Table 3). Having ever been diagnosed with hepatitis C was reported by 31%, with 30% reporting an asthma diagnosis, and 11% reporting emphysema or COPD diagnosis (Table 3).

### Service use

When asked about contact with a range of health and social welfare services, only 4% said they had not used any services in the past 6 months (Table 3), and 69% said they had been in contact with a specialist drug service and 63% a general practitioner (primary care doctor) in the past 6 months.

Almost two-thirds (62%) were receiving OST (Table 3). Stratifying this by route of administration, 59% (*n* = 301/509) who reported smoking crack-cocaine but not injecting drugs were receiving OST, compared to 68% (149/219) of those reporting injecting (*p* = 0.023). Among those on OST the use of sedative drugs in past 28 days was common: with 84% (377/450) using heroin, 41% (184/450) pregabalin/gabapentin, and 29% (132/450) benzodiazepines, with 17% (77/450) of those on OST reporting use of all three sedative drug types (i.e., heroin, pregabalin/gabapentin, and benzodiazepines).

### Gender differences

Women were younger than the men (41.2 years vs. 42.4 years) and were less like to have hand contact with the criminal justice system (e.g., 55% vs 78% had ever been imprisoned) (Table 1). They were more likely to report sex work (30% vs 1%) and less likely to report temporary work (5% vs 11%, Table 1). On average women started using crack-cocaine at an older age (23.9 years vs. 21.8 years) and had been using for fewer years (17.6 years vs. 20.6 years) than the men (Table 2). Women tended to use crack-cocaine at a friend’s house more often than the men did, and to use more often in the company of a partner, a dealer, or sex work client (Table 2). Fewer women reported using cannabis, cocaine, spice/synthetic cannabinoids or amphetamines than men, but women more often reported using vapes or e-cigarettes (Table 2).

Women were more likely to report having a respiratory symptom that they associated with their crack-cocaine use (i.e., problem with breathing, chest pain, coughing blood or phlegm) than men (67% vs 58%, Table 3). They were also more likely to have ever been hospitalised with pneumonia (25% vs 14%) and have ever been diagnosed with asthma, emphysema or COPD, and hepatitis C (Table 3). Ambulance use, hospital admission, and use of a sex worker support service in the past 6 months were more commonly reported by women than men (Table 3).

### Factors associated with symptoms of crack-related respiratory problem by gender

The factors associated with having a symptom of a crack-related respiratory problem among both the women and men in the bivariate analyses are presented in Table 4. Among both men and women there were similar associations with age at first crack-cocaine use, time since first crack-cocaine use, pipe sharing, and the places where crack-cocaine had been used and reporting a symptom of a crack-related respiratory problem. However, among women having a symptom of a crack-related respiratory problem was associated with injecting drugs during the past 28 days, unlike for men. Among the men having a symptom was associated with income sources, and using with casual friend/acquaintance, but not for women. The associations with the other drugs used varied with gender (Table 4).

**Table 4:** Associations between socio-demographic characteristics, drug use and service use with reporting a crack-cocaine related'respiratory symptom', by gender

		**Crack-related respiratory symptom (i.e., problem with breathing, chest pain, coughing blood or phlegm)**											
		**Women**						**Men**					
		**No**		**Yes**		**Total**	*p*	**No**		**Yes**		**Total**	*p*
		69	33%	138	67%	207		217	42%	297	58%	514	
*Socio-demographics*													
Age, years	Mean	41.38		41.22		41.3		42.06		42.60		42.4	
	Median	41.00		41.00		41	0.704^‖^	42.00		43.00		42	0.325^‖^
	Range (Q25, Q75)	34 (35, 46)		42 (36, 45)		42 (36, 46)		46 (37, 47)		52 (37, 48)		53 (37, 48)	
Ethnicity~	Minority	8	12%	19	14%	27	0.648	29	13%	39	13%	68	0.962
	White	61	88%	118	86%	179		188	87%	256	87%	444	
	*Total*	*69*		*137*		*206*		*217*		*295*		*512*	
Sexual orientation*	Heterosexual	53	78%	95	71%	148	0.321	206	95%	282	97%	488	0.490
	Gay, lesbian, bisexual or in another way	15	22%	38	29%	53		10	5%	10	3%	20	
	*Total*	*68*		*133*		*201*		*216*		*292*		*508*	
Housing status	Homeless/rough sleeper	9	13%	21	15%	30	0.321	40	18%	58	20%	98	0.053
	Unstable ^†^	17	25%	34	25%	51		49	23%	82	28%	131	
	Stable ^‡^	43	62%	81	59%	124		125	58%	143	48%	268	
	Other/refuse**	0	0%	2	1%	2		3	1%	14	5%	17	
How did you get by in the past 6 months?	Held a regular job (full or part-time)	5	7%	5	4%	10	0.306^¶^	15	7%	16	5%	31	0.473
	Benefits	61	88%	128	93%	189	0.295	196	90%	262	88%	458	0.449
	Temporary work	5	7%	5	4%	10	0.306^¶^	16	7%	42	14%	58	0.017
	Borrowed/got money from family or friends	28	41%	73	53%	101	0.095	87	40%	152	51%	239	0.013
	Sold sex ^§^	18	26%	45	33%	63	0.336	1	0%	4	1%	5	0.403^¶^
	Sold drug	10	14%	26	19%	36	0.437	31	14%	44	15%	75	0.867
	Theft/burglary	13	19%	32	23%	45	0.475	40	18%	64	22%	104	0.385
	Begged	17	25%	42	30%	59	0.384	58	27%	112	38%	170	0.009
	Other(s)/None of the above	2	3%	3	2%	5	1.000^¶^	11	5%	11	4%	22	0.450
Can you always access food when you need it?		46	67%	100	74%	146	0.305	168	77%	205	70%	373	0.053
	*Total*	*69*		*136*		*205*		*217*		*294*		*511*	
Contact with criminal justice system													
Have you ever been in prison or a young offenders' institution?		35	51%	80	58%	115	0.295	158	74%	238	80%	396	0.092
	*Total*	*69*		*137*		*206*		*214*		*297*		*511*	
In the last six months, did the police stop and search you?		18	26%	48	35%	66	0.182	81	38%	128	43%	209	0.192
	*Total*	*69*		*136*		*205*		*216*		*296*		*512*	
In the last six months, did the police take away your smoking kit or any other drug equipment?		8	12%	15	11%	23	0.918	42	19%	63	21%	105	0.611
	*Total*	*69*		*135*		*204*		*216*		*296*		*512*	
*Crack* *use*													
How old were you when you first started using crack? (years)	Mean	26.58		22.66		24.0		23.00		20.10		21.8	
	Median	26.00		20.50		22	0.001^‖^	21.00		19.00		20	0.001^‖^
	Range (Q25, Q75)	38 (19, 32)		43 (17, 28)		45 (17, 30)		45 (17, 28)		49 (16, 24)		50 (16, 26)	
	*Total*	*67*		*136*		*203*		*216*		*297*		*513*	
Time since first crack use, in years	Mean	15.12		18.71		18		19.02		21.58		20.50	
	Median	16.00		21.00		19	0.012^‖^	20.00		23.00		22	0.005^‖^
	Range (Q25, Q75)	35 (6, 22)		41 (11, 25)		42 (9, 25)		47 (10, 26)		51 (15, 29)		51 (13, 28)	
	*Total*	*67*		*136*		*203*		*216*		*297*		*513*	
Smoked or injected crack daily, past 28 days		38	55%	85	62%	123	0.368	114	53%	160	54%	274	0.764
Frequency of crack use past 28 days, in days	Mean	20.8		22.9		22.2		20.2		21.4		20.9	
	Median	28		28		28	0.198^‖^	28		28		28	0.304^‖^
	Range (Q25, Q75)	27 (14, 28)		27 (20, 28)		27 (20, 28)		27 (12, 28)		27 (15, 28)		27 (14, 28)	
Injected crack, past 28 days		10	14%	45	33%	55	0.005	68	31%	91	31%	159	0.866
Injected crack with heroin in past 28 days, all participants		9	13%	42	30%	51	0.006	66	30%	84	28%	150	0.599
Used homemade pipe, among those smoking past 28 days.		39	57%	95	69%	134	0.080	146	67%	217	73%	363	0.155
Shared pipes, among those smoking past 28 days.		19	28%	71	51%	90	0.001	83	38%	145	49%	228	0.017
Places where crack was used past 28 days (among those smoking past 28 days)	At home	58	84%	102	74%	160	0.100	149	69%	223	75%	372	0.108
	At work	4	6%	2	1%	6	0.097^¶^	6	3%	5	2%	11	0.540^¶^
	At a friend's place	26	38%	73	53%	99	0.039	59	27%	118	40%	177	0.003
	Outdoors	27	39%	83	60%	110	0.004	98	45%	167	56%	265	0.013
	In a semi-public space (e.g., stairwell)	17	25%	51	37%	68	0.075	52	24%	103	35%	155	0.009
	Abandoned building	8	12%	35	25%	43	0.021	35	16%	86	29%	121	0.001
	Somewhere else	5	7%	21	15%	26	0.103	18	8%	26	9%	44	0.854
Smoked crack with (among those smoking past 28 days):	Partner	28	41%	56	41%	84	1.000	34	16%	53	18%	87	0.516
	Other family member	4	6%	14	10%	18	0.295	17	8%	22	7%	39	0.857
	Close friend	23	33%	46	33%	69	1.000	90	41%	101	34%	191	0.084
	Housemates/co-residents	11	16%	19	14%	30	0.675	24	11%	39	13%	63	0.479
	Dealer/runner	7	10%	13	9%	20	0.868	10	5%	13	4%	23	0.900
	Casual friend/acquaintance	29	42%	57	41%	86	0.921	78	36%	145	49%	223	0.004
	Client (e.g. paying sex partner)	7	10%	5	4%	12	0.110^¶^	1	0%	1	0%	2	1.000^¶^
	Sex worker	4	6%	4	3%	8	0.445^¶^	5	2%	9	3%	14	0.617
	Someone else	3	4%	9	7%	12	0.754^¶^	6	3%	14	5%	20	0.259
*Use of other drugs/substances*													
Substances used past 28 days	Heroin	53	77%	110	80%	163	0.631	182	84%	215	72%	397	0.002
	Cannabis/weed	35	51%	76	55%	111	0.554	141	65%	195	66%	336	0.873
	Pregabalin/gabapentin	21	30%	75	54%	96	0.001	71	33%	131	44%	202	0.009
	Benzodiazepines	11	16%	43	31%	54	0.019	51	24%	93	31%	144	0.051
	Cocaine	13	19%	30	22%	43	0.628	60	28%	91	31%	151	0.462
	Spice/synthetic cannabinoids	7	10%	22	16%	29	0.257	64	29%	88	30%	152	0.973
	Amphetamines (speed)	2	3%	7	5%	9	0.721^¶^	27	12%	28	9%	55	0.275
	Ketamine	3	4%	9	7%	12	0.754^¶^	13	6%	27	9%	40	0.195
	Mephedrone/M-cat	6	9%	9	7%	15	0.570	16	7%	20	7%	36	0.779
	Tramadol	4	6%	10	7%	14	0.778^¶^	15	7%	18	6%	33	0.697
	Crystal meth	1	1%	1	1%	2	1.000^¶^	4	2%	14	5%	18	0.080
	GHB/GBL/G	0	0%	3	2%	3	0.552^¶^	4	2%	6	2%	10	1.000^¶^
	Alcohol	36	52%	82	59%	118	0.321	110	51%	189	64%	299	0.003
	Tobacco	61	88%	124	90%	185	0.750	184	85%	280	94%	464	<0.001
	Vapes/E-cigs	29	42%	69	50%	98	0.279	73	34%	123	41%	196	0.073
Injected drug past 28 days		15	22%	49	36%	64	0.043	76	35%	94	32%	170	0.422

Those with symptoms of a crack-related respiratory problem reported having breathing problems, chest pain, and/or coughing up blood/phlegm that they thought was related to smoking crack-cocaine

A total is given for an item when this differs from 721 due to people declining to answer

~ Ethnic minority refers to all ethnic groups excluding White only ethnicities. These include Asian/British Asian, Black/Black British, Arah, Hispanic/Latino, and ‘other’ ethnicities

† Unstable includes hostel, squat, prison, rehab, staying friends & family ('sofa surfing')

‡ Stable includes having own place, and being in a house share

§ Sold sex, any sexual services i.e. oral, vaginal, anal, etc.

‖ Mann-Whitney U test

¶ Chi-squared using Fisher's Exact Test

* Gay, lesbian, or bisexual incl. 4 reporting other sexual orientations; 12 missing (don't know=6, refuse=6)

** Excluded from the analysis for women

In the multivariable analysis (Table 5) having a symptom of a crack-related respiratory problem among men was associated with: increasing time since first used crack-cocaine; food insecurity; current use of pregabalin/gabapentin and tobacco; temporary employment; and crack-cocaine use in abandoned buildings or at friend’s place. Current use of heroin and using with a close friend were both associated with reduced odds of having a respiratory symptom. Among women, having a symptom of a crack-related respiratory problem was also associated with a longer time since first crack-cocaine use and current use of pregabalin/gabapentin, but additionally with sharing pipes (Table 5). Those with symptoms of a crack-related respiratory problem reported having breathing problems, chest pain, and/or coughing up blood/phlegm that they thought was related to smoking crack-cocaine

**Table 5:** Multivariable analysis of associations between socio-demographic characteristics, drug use and service use with reporting a crack related 'respiratory symptom', by gender

		**Crack related respiratory symptom**							
		**Yes**		**Total**	*P*	**Adjusted Odds Ratio**	**95% CI Adjusted Odds Ratio**		
*Men*									
Time since first crack use, in years				507	0.009	1.03	1.01	-	1.05
Can you always access food when you need it?	No	89	65%	136	0.034	1.61	1.04	-	2.49
	Yes	205	55%	371		1.00			
Pregabalin/gabapentin used in past 28 days	No	163	53%	307		1.00			
	Yes	131	66%	200	0.039	1.53	1.02	-	2.29
Tobacco used in past 28 days	No	17	35%	49		1.00			
	Yes	277	60%	458	<0.001	3.57	1.81	-	7.02
Heroin used in past 28 days	No	80	70%	114		1.00			
	Yes	214	54%	393	<0.001	0.32	0.19	-	0.54
Did temporary work	No	252	56%	449		1.00			
	Yes	42	72%	58	0.033	1.99	1.06	-	3.76
Smoked at a friend's place	No	178	53%	334		1.00			
	Yes	116	67%	173	0.019	1.70	1.09	-	2.65
Smoked in abandoned building	No	208	54%	386		1.00			
	Yes	86	71%	121	0.037	1.69	1.03	-	2.76
Smoked with a close friend	No	194	61%	317		1.00			
	Yes	100	53%	190	0.007	0.57	0.38	-	0.86
*Women*									
Time since first crack use, in years				203	0.024	1.04	1.01	-	1.08
Shared pipes past 28 days	No	66	57%	115		1.00			
	Yes	70	80%	88	0.001	3.04	1.57	-	5.90
Pregabalin/gabapentin used in past 28 days	No	62	57%	108		1.00			
	Yes	74	78%	95	0.009	2.35	1.23	-	4.47

All variables included in the final models are presented in the table

Those with symptoms of a crack-related respiratory problem reported having breathing problems, chest pain, and/or coughing up blood/phlegm that they thought was related to smoking crack-cocaine

## Discussion

This study, which was focused on those using crack-cocaine by any route, indicates that people using crack-cocaine in England experience high levels of respiratory health harm, and there are variations in use and harm by gender. Previous UK surveys that have looked at crack-cocaine use have examined this in samples of people who are injecting drugs or who are using opioids [10–12], yet in our study 99% smoked crack-cocaine and 30% of participants injected it. Poly-drug use was very common, with four-fifths also reporting use of heroin, two-thirds cannabis, and two-fifth pregabalin and/or gabapentin. There were also high levels of homelessness, engagement with the criminal justice system, and contact with health services.

It is important to consider the potential limitations of the data presented, as these are taken from a baseline survey for an intervention study which recruited participants at six locations across three regions of England. These locations included a range of urban, town and more rural settings, with participants recruited through a structured sampling approach that aimed to capture a broadly representative sample of those who use crack-cocaine. While the sample is likely to be broadly representative of the study sites caution should be taken when generalising the findings to the rest of England and the UK. The survey questions were based on ones that have been used previously and refined with community input, however, the measures utilised are self-reports which may be subject to recall or social desirability bias, and the health symptoms and outcomes were not clinically validated. The measure of crack-related respiratory symptoms used is based on questions that ask about experiences of a range of symptoms of varying severity that they related to their crack-cocaine use, when interpreting the findings related to this measure the broad spectrum of symptoms included, and that these are self-reported, should be considered. However, self-reported behaviours among people who use drugs are generally reliable, particularly in computer-assisted surveys [46, 47]. Trans participants were included in the analysis according to their identified gender. However, the gender analysis was binary (women/men) due to small numbers of trans and non-binary participants. This resulted in the exclusion of the ‘non-binary’ category (*n* = 1). Finally, this was a cross-sectional study focused on exploring crack-cocaine use, and so does not allow us to examine causation.

The high levels of contact with the criminal justice system and unstable housing found here are similar to those amongst people who use heroin or inject in the UK [10, 12], and are unsurprising considering the marginalisation and illicit nature of crack-cocaine use. Overall, this study confirms that this population is financially insecure, unstably housed, and criminalised, with these environmental stresses likely to compound risk (e.g., from using improvised pipes due to a lack resources to purchase pipes) and exacerbate poor health (e.g., the impact of living on the street or in damp housing on respiratory problems).

Those using crack-cocaine were also using a wide range of other drugs, reflecting complex and varied patterns of poly-drug use, with significant differences by gender. This aligns with recent indications of increased poly-drug use among those who inject drugs and/or use opioids in the UK and the rest of Europe [12, 48]. Poly-sedative use, which elevates the risk of fatal overdose, was especially common across the sample. Opioids act as a depressant on the central nervous system which, in the case of an overdose, depresses respiratory functioning and can lead to complete respiratory failure. Due to similar pharmacodynamic processes, combining opioids with other sedatives, such as alcohol, benzodiazepines, and pregabalin, increases the potential of respiratory crisis and death [49–51]. The potential impacts of the use of multiple respiratory depressants on morbidity and mortality among those using crack-cocaine is a concern considering the extent respiratory health problems in this population.

Almost a third reported having been diagnosed with asthma and one in six had ever been admitted to hospital with pneumonia, with a quarter women having been admitted to hospital with pneumonia compared to only one-in-seven of men. Overall, three-fifths reported experiencing a respiratory symptom that they related to crack-cocaine smoking. In both men and women having respiratory symptom was associated with longer time since first crack-cocaine use and the use of pregabalin/gabapentin which are respiratory depressants [52]. It should be noted that crack-cocaine was not the only substance being smoked, with the smoking of tobacco, cannabis and synthetic cannabinoids common and many will have also smoked heroin. Smoking multiple drugs has additive effects on respiratory health [53–55], and therefore likely contributes to some of these symptoms. This would appear to be the case for men as smoking nicotine was associated with experiencing a respiratory symptom in the multivariable analysis. However, crack-cocaine smoking may pose a particular risk to respiratory health, as women experienced higher prevalences of respiratory symptoms, diagnoses and hospitalisations than men, but used both cannabis and synthetic cannabinoids—which are usually smoked—much less often than men. Poor respiratory health could also have been impacted by sleeping on the streets and living in poor quality housing, which were common.

The smoking of crack-cocaine is, however, likely to play a major role in causing of these symptoms due to the dangers that are inherent in smoking crack-cocaine using homemade devices [9, 45, 56–58]. These ‘homemade’ smoking devices can lead to the inhalation of very hot vapours, particulates from the media used to support the burning crack-cocaine (such as ash or wire mesh), and noxious gases from improvised pipes made of plastic or metal (e.g., made from asthma inhalers or metal tubing) [56]. Due to the lack of legal supply of smoking equipment, people who use crack-cocaine in the UK report using such homemade smoking devices, [9, 56] thus indicating a need for interventions to reduce risk of respiratory harms from crack-cocaine smoking using unsafe improvised devices.

Symptoms of respiratory problems that participants related to crack-cocaine smoking were less common among men than women, even so, three-fifths of the men reported recently experiencing a respiratory symptom. These symptoms in men appear to be related to the impact of socio-environmental instability, as having symptoms was associated with difficulty accessing food, temporary work, and smoking at a friend's place or in an abandoned building, with heroin use (the focus of treatment and support services in the UK) and smoking with a close friend (a source of social support) being protective.

Women reported higher levels of symptoms, and being diagnosed with asthma, emphysema or COPD more often than men, which may reflect gender related differences in susceptibility or severity [59, 60]. On average women in this study had started to use crack-cocaine at an older age, and overall had been using crack-cocaine for fewer years, than the men, suggesting that they may be developing these respiratory symptoms and conditions more quickly than men. This aligns with the wider COPD literature, as diagnosis for COPD normally occurs at lower levels of tobacco exposure for women compared to men [61, 62]. This may reflect the ‘telescoping’ effect that has been reported in some studies globally where the development of problematic drug use and drug related harms appear to happen more rapidly among women than men [63]. There is evidence from a wide range of studies of this telescoping effect among women for a variety of drugs, including stimulants, that indicate whilst women start use latter they experience an accelerated course to dependant and problematic use [63]. This could reflect the gendered power dynamics that can contribute to higher levels of marginalisation and harms that can be experienced by women who use drugs [26, 27, 30–33]. These gendered differences can occur as result of the social contexts of crack-cocaine use at the micro level (e.g., male partners sourcing drugs and pipe sharing with partners) and at the meso level (e.g., impact of local policing practices related to drug use and sex work), as well as a consequence of gendered structural vulnerabilities at macro level (e.g., societal issues that lead to sex work and financial insecurity) [64, 65]. Alternatively, this could reflect biological differences, such as anatomy or hormonal factors, that affect susceptibility to harm [66–68]. However, the differences found here will probably reflect an interplay between these issues, and thus the gender differences in extent of respiratory problems, and in particular these being more common and possibly occurring more rapidly among women, need further investigation.

## Conclusion

In the UK service provision for people who use drugs is focused on opiate use and injecting, however, the use of crack-cocaine has become more common. Crack-cocaine is most often smoked, but currently UK services have little to offer those who smoke crack-cocaine. Interventions for those who smoke crack-cocaine in the UK are needed, and considering the extent of respiratory harms, the provision of safer pipes needs to be considered as this is likely to reduce harms and support service engagement [69]. Interventions to promptly detect and respond to respiratory damage among people smoking crack-cocaine, and other drugs, need to be developed Responses to address the gender related differences in crack-cocaine related harms, particularly the higher levels of respiratory harm among women, are needed. This could include developing more gender inclusive services for people who use drugs and approaches to reach out to women who may be reluctant to access services, such as, through the provision of targeted and peer outreach services.

## Data Availability

Deidentified datasets will be stored on LSHTM's data repository and requests for access will be considered and where appropriate made available http://datacompass.lshtm.ac.uk

## Abbreviations

BBV: Blood borne virus
COPD: Chronic obstructive pulmonary disease
IQR: Interquartile range
MDA: Misuse of Drugs Act
NSP: Needle and syringe programme
OST: Opioid substitution therapy
SIPP: Safe Inhalation Pipe Provision
UK: United Kingdom
